# Self-emulsified annatto tocotrienol improves bone histomorphometric parameters in a rat model of oestrogen deficiency through suppression of skeletal sclerostin level and RANKL/OPG ratio

**DOI:** 10.7150/ijms.64045

**Published:** 2021-09-09

**Authors:** Nur-Vaizura Mohamad, Soelaiman Ima-Nirwana, Kok-Yong Chin

**Affiliations:** Department of Pharmacology, Faculty of Medicine, Universiti Kebangsaan Malaysia, 56000 Cheras, Kuala Lumpur, Malaysia.

**Keywords:** menopause, oestrogen deficiency, osteopenia, sclerostin, skeleton, vitamin E

## Abstract

Menopause is the leading cause of osteoporosis for elderly women due to imbalanced bone remodelling in the absence of oestrogen. The ability of tocotrienol in reversing established bone loss due to oestrogen deficiency remains unclear despite the plenitude of evidence showcasing its preventive effects. This study aimed to investigate the effects of self-emulsified annatto tocotrienol (SEAT) on bone histomorphometry and remodelling in ovariectomised rats. Female Sprague Dawley rats (n=36) were randomly assigned into baseline, sham, ovariectomised (OVX) control, OVX-treated with annatto tocotrienol (AT) (60 mg/kg), SEAT (60 mg/kg) and raloxifene (1 mg/kg). Daily treatment given through oral gavage was started two months after castration. The rats were euthanised after eight weeks of treatment. Blood was collected for bone biomarkers. Femur and lumbar bones were collected for histomorphometry and remodelling markers. The results showed that AT and SEAT improved osteoblast numbers and trabecular mineralisation rate (p<0.05 vs untreated OVX). AT also decreased skeletal sclerostin expression in OVX rats (p<0.05 vs untreated OVX). Similar effects were observed in the raloxifene-treated group. Only SEAT significantly increased bone formation rate and reduced RANKL/OPG ratio (p<0.05 vs untreated OVX). However, no changes in osteoclast-related parameters were observed among the groups (p>0.05). In conclusion, SEAT exerts potential skeletal anabolic properties by increasing bone formation, suppressing sclerostin expression and reducing RANKL/OPG ratio in rats with oestrogen deficiency.

## Introduction

Postmenopausal women are the population most affected by osteoporosis, a systemic skeletal disease featuring bone density and microarchitecture degeneration, resulting in decreased bone strength and fragility fractures 1. Multiple epidemiological studies indicated that menopause is a significant predictor of osteoporosis in women 2-4. Osteoporosis and its complications are significant health issues worldwide due to the growing healthcare costs and mortality post-fracture in line with the expanding geriatric population 5-8.

Bone remodelling is a continuous reparative process to preserve skeletal integrity and mineral homeostasis. It is regulated by osteoclasts, osteoblasts and osteocytes through a complex network of signalling pathways and control mechanisms. Several hormones [e.g. sex hormones and parathyroid hormone (PTH)], osteoblast/osteocyte/bone marrow-derived cytokines and growth factors are involved in bone health maintenance 9-11. Receptor activator of nuclear factor kappa-B (RANK) ligand (RANKL) is a crucial stimulator for osteoclastogenesis by binding to RANK receptors on osteoclast precursor cells 12. Osteoprotegerin (OPG) acts as a decoy receptor of RANKL by preventing the binding between RANK and RANKL. Both cytokines are secreted by osteoblast lineage cells 13, 14. Persistent elevation of PTH (as in calcium deficiency) causes skeletal catabolic effects by elevating RANKL expression and lowering OPG expression of osteoblasts 15, 16. Thus, the RANKL/OPG ratio is indicative of the balance between bone resorption and formation. The canonical Wnt/β catenin signalling stimulates osteoblast maturation and survival while inhibits osteoclast formation by upregulating OPG expression by osteoblasts/osteocytes 17, 18. Osteocytes antagonise Wnt signalling by releasing Dickkopf protein-1 (DKK-1) and sclerostin (SOST) 19-21. Oestrogen production ceases during the menopausal transition, leading to excessive bone resorption and inadequate bone formation due to the disturbance of the pathways mentioned.

Tocotrienol, a member of the vitamin E family, can be found in annatto bean, palm oil and rice bran. Tocotrienol shares a similar structure with tocopherol, consisting of a chromanol head and a long carbon tail. Tocotrienol has three double bonds on the carbon tail, while tocopherol has only single bonds. The position/number of methyl groups on the chromanol ring further divides tocotrienol into alpha-, beta, gamma- and delta-isomers. Tocotrienol from annatto bean consists of predominantly delta-tocotrienol (~90%) and some gamma-tocotrienol (~10%) without tocopherols 22. This unique composition allows researchers to examine the biological action of a natural tocotrienol composition without the influence of tocopherols. Previous studies demonstrated that annatto tocotrienol (AT) [60 mg/kg body weight (b.w.)] could prevent bone loss due to oestrogen deficiency in rats by upregulating bone morphogenetic protein-2 mRNA expression 23-25. In studies involving surgical/chemical-induced testosterone deficit male rats, AT (60 mg/kg b.w.) preserved bone health by increasing expression of genes coding beta-catenin, type-2 collagen and alkaline phosphatase and decreasing RANKL mRNA expression 26-28. Our previous study has shown that AT formulated with a self-emulsifying drug delivery system increased the plasma delta-tocotrienol by 4 folds and preserved bone structure calcium content and strength in rats with oestrogen deficiency 29. The role of osteocyte-related factors in the mechanism of skeletal protection for AT in rats with prolonged oestrogen deficiency remains elusive.

The objective of this study was to determine the effects of self-emulsified AT (SEAT) on bone histomorphometry and remodelling factors in rats with prolonged oestrogen deficiency, focusing on osteocyte-related factors. It was hypothesised that SEAT could alter bone remodelling factors associated with osteocytes in rats with prolonged oestrogen deficiency, and the effects would be translated in bone histomorphometric indices.

## Materials and methods

### Preparation of SEAT

AT (84% delta-tocotrienol and 16% gamma-tocotrienol) was provided by American River Nutrition Inc. (Hadley, MA, US). The self-emulsifying system comprises 40.7% w/w primary surfactant (Cremophor® EL, International Lab, San Francisco, USA), 40.7% w/w co-surfactant (Labrasol®, International Lab, San Francisco, USA), 7.2% w/w oil (Captex® 355, International Lab, San Francisco, USA) and 11.4% w/w ethanol as co-solvent (HmBG, Hamburg, Germany) 30. SEAT was prepared by adding 35% w/w AT to the self-emulsifying system and vortexing the mixture for ~2 min until a homogenous solution was obtained. Vitamin E-stripped corn oil (Dyets Incorporated, Bethlehem, USA) was used to dilute AT and SEAT. The treatment was given for eight weeks via oral gavage daily at a dose of 60 mg/kg b.w.

### Study design

Universiti Kebangsaan Malaysia Animal Ethics Committee reviewed and approved the study protocol (UKMAEC Approval code: FAR/PP2018/IMA NIRWANA/23-JAN./898-MAR.-2018-/FEB.-2020). The institutional and national guidelines for animal handling and care were adhered to during the study. Female Sprague-Dawley rats aged ten-month-old (n=36, 200-250 g) were obtained from the Laboratory Animal Resource Unit, Faculty of Medicine, Universiti Kebangsaan Malaysia, Kuala Lumpur, Malaysia. The rats were placed in an animal housing facility with the following conditions: two animals per plastic cage, temperature 27±2 °C, 10/14 hours light-dark cycle, unrestricted access to commercial rat chow (720-P, Gold Coin, Klang Selangor, Malaysia) and tap water. Randomisation into experimental groups (n=6/group) was performed after seven days of acclimatisation. The baseline (BSL) group was sacrificed without any interventions. Bilateral ovariectomy was performed on all other groups except the sham-operated (SHAM) group under anaesthesia (ketamine/xylazine). Laparotomy was performed on the SHAM group without removing the ovaries to induce similar surgical stress as the ovariectomised groups. Baytril® 5% (5 mg/kg b.w.) (Bayer Health Care, Leverkusen, Germany) was administrated intramuscularly for five days post-surgery to prevent infection. AT, SEAT and raloxifene treatments were initiated eight weeks after ovariectomy to allow the rats to recuperate and develop bone loss. The SHAM and ovariectomised control (OVX) groups were given the blank self-emulsifying system by oral gavage. The rest were given AT (60 mg/kg b.w. orally daily), SEAT (60 mg/kg b.w. orally daily) or raloxifene hydrochloride (1 mg/kg b.w. orally daily; International Lab, San Francisco, USA) (RAL group). The rats were euthanised with cardiac puncture under anaesthesia (ketamine/xylazine) and the bones were harvested for analysis. Blood samples were collected using plain tubes (BD Vacutainer®SSTTM Advance). Serum was separated by centrifugation at 3000 rpm for 10 min and stored at -70 °C for bone biomarkers analysis.

### Measurement of bone histomorphometry

The right femur was fixed for 72 hours in 70% formaldehyde. The distal third was divided sagittally using a low-speed saw (RTX-1 Rotary Tool, Black & Decker, Baltimore, USA). The decalcification of the first half was performed using EDTA solution (Sigma-Aldrich, St Louis, MO, USA) for eight weeks. The second half was placed in phosphate buffer formalin before being embedded in polymerised resin (Catalog no: EM0200 Osteo-Bed Bone embedding kit, Sigma-Aldrich, St Louis, MO, USA). The undecalcified bones samples were sectioned (Leica TC-65 carbide metal disposable blades) at a thickness of 9 μm using a microtome (Leica RM2235, Nussloch, German). The unstained sections were used to assess bone dynamic parameters. The decalcified samples were embedded in paraffin (Leica Biosystems, Richmond Inc, USA) and sectioned (Leica 818 high profile microtome blade) at a thickness of 5 μm using the same microtome (Leica RM2235, Nussloch, German). Sections were stained using a hematoxylin-eosin (H&E) kit (Catalog no; ab245880, Abcam, Cambridge, UK) to assess bone cellular parameters. The bone histological sections were viewed under white light and analysed using an image analyser (Olympus BX53 Upright Microscope, Japan). The dynamic parameters were viewed under a fluorescence microscope (Nikon Eclipse 80i, Tokyo, Japan) and measured manually using Image J (Java-based, National Institutes of Health, Maryland, USA) and Weibel grid technique 31, 32.

**Figure 1:**
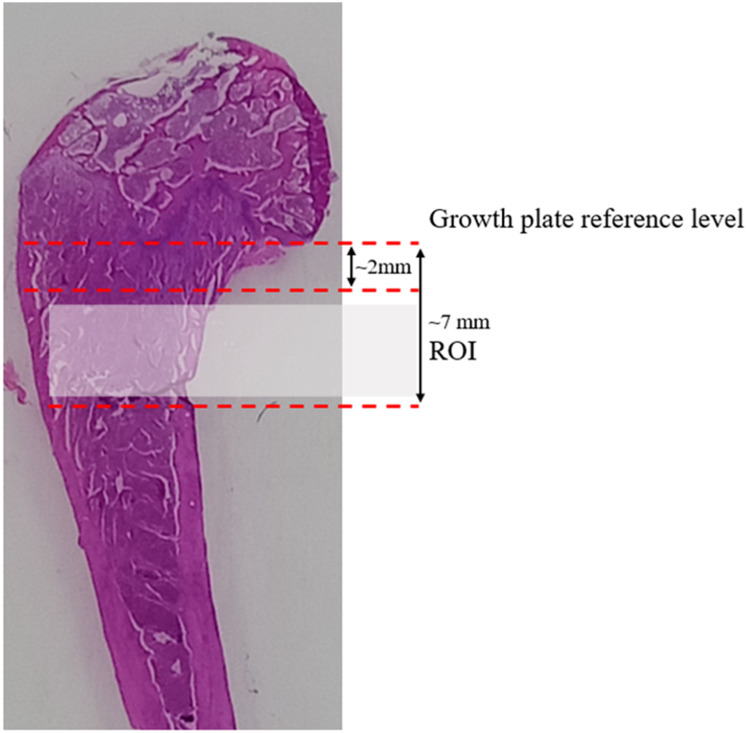
The distal femoral longitudinal section shows the margin for sampling in the metaphyseal area. The metaphyseal area was located ~ 2-7 mm from the lowest point of the growth plate.

Secondary spongiosa of the metaphyseal region, located ~2-7 mm from the lowest point of the femoral growth plate, was examined for bone histomorphometry (Fig. 1). Four sequential slides were examined for each rat. Dynamic histomorphometric measurements were determined using double fluorescent labelling technique with an intraperitoneal calcein injection (20 mg/kg b.w., Sigma-Aldrich, St Louis, MO, USA) at nine and two days before the sacrifice. The parameter examined were single labelled surface over bone surface (sLS/BS, unit=%) and double-labelled surface over bone surface (dLS/BS, unit=%). The other parameters derived are mineralizing surface over bone surface (MS/BS, unit=%) = [dLS + 1/2 sLS]/BS and bone formation rate over bone surface (BFR/BS, unit=μm3/μm2/day) = MS/BS*MAR. The mineral appositional rate (MAR, unit=µm/day), was the mean distance between two labels over seven days. The cellular histomorphometric parameter measured included osteoblast surface over bone surface (ObS/BS, unit=%), osteoclast surface over bone surface (OcS/BS, unit=%), eroded surface over bone surface (ES/BS, unit=%), osteoid surface over bone surface (OS/BS, unit=%) and osteoid volume over bone volume (OV/BV, unit=%), osteocyte surface over bone surface (OtS/BS, unit=%), empty lacunae surface over bone surface (ELc/BS, unit=%) and total lacunae surface over bone surface (TtLc/BS, unit=%).

### Measurement of bone biomarkers

Serum osteocalcin (bone formation marker; catalogue no: E-EL-R0243) and beta C-terminal telopeptide (β-CTX, bone resorption marker; catalogue no: E-EL-R1405) levels were determined using commercial quantitative sandwich enzyme-linked immunosorbent assay kits specific for rats (Elabscience® Biotechnology Inc, Wuhan, China) and the absorbance was read with EnSpire® Multimode Microplate Reader (PerkinElmer, Hopkinton, MA, USA). The protocols were per the instructions of the manufacturer.

### Measurement of bone remodelling markers

The fourth lumbar, cleaned of soft tissues, was weighed and homogenised using mortar and pestle in liquid nitrogen. Proteins in the homogenized bone were extracted using a buffer containing 50 mM Tris (pH 7.4), 150 mM sodium chloride (NaCl), 1% Triton X-100, 1% sodium deoxycholate, 1 mM ethylenediaminetetraacetic acid, 0.1% sodium dodecyl sulphate, 10 nM sodium fluoride, 1 mM sodium orthovanadate, and 1 mM phenylmethylsulfonyl fluoride (Elabscience Biotechnology Houston, China). The supernatant was collected after centrifugation (8000 g for 10 minutes) for total protein quantification using the Bradford method (Quick StartTM Bradford Protein Assay, Bio-Rad Laboratories Inc, Hercules, USA). The level of bone remodelling regulatory proteins (DKK-1, SOST, OPG, PTH) in the fourth lumbar were determined using Milliplex® Map Kits (EMD Millipore Corporation, Billerica, USA) according to the manufacturer's instructions, and the absorbance was read with Luminex 100 (Luminex Corporation, Austin, USA). Soluble RANKL (sRANKL) level in the lumbar samples was determined using enzyme-linked immunosorbent assay kits according to the manufactures' instructions (Fine Test, Wuhan, China). The target protein levels were normalised with the total protein concentration of each sample.

### Statistical analysis

Shapiro-Wilk test was used to assess the normality of the data, and they are normally distributed. One-way analysis of variance (ANOVA) with post hoc pairwise (Tukey or Dunnett T3) comparison was used to compute the differences in bone histomorphometric parameters, circulating bone remodelling and skeletal regulatory protein levels. Statistical significance was considered at p<0.05. The data were expressed in mean ± standard error of the mean. Statistical Package for Social Sciences (SPSS) version 22.0 (IBM, Armonk, USA) was used in the statistical analysis.

## Results

**Figure 2:**
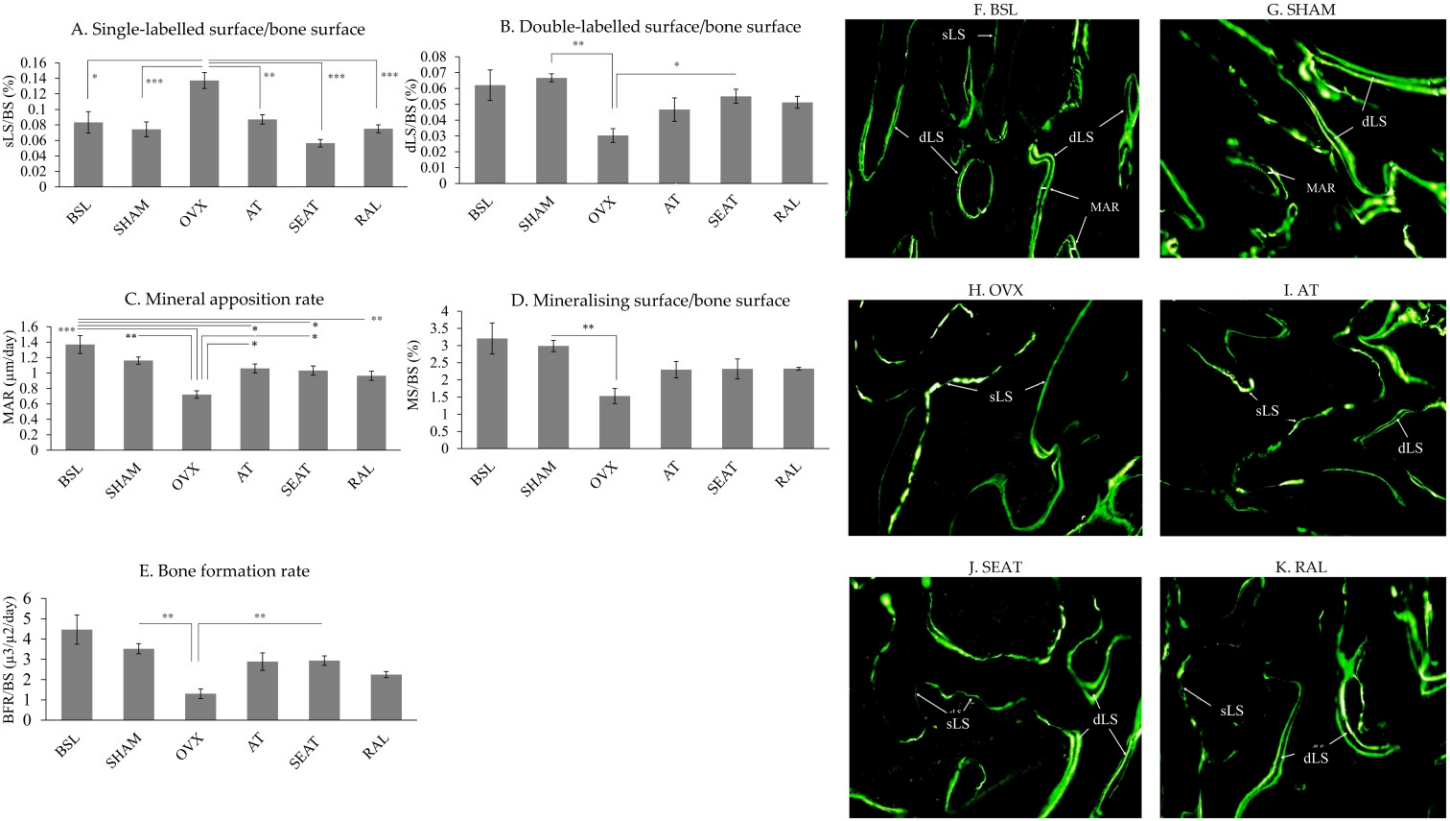
The mineralisation activity of trabecular bone at the distal femur is assessed via bone dynamic histomorphometric indices (A-E). The data are expressed in mean ± standard error of the mean. *, ** and *** indicate statistically significant differences at p<0.05, p<0.01 and p<0.001. Photomicrograph (F-K) shows calcein labels at the trabecular bone of distal femur, demonstrated using fluorescence microscopy in an undecalcified section without staining in the six study groups (20 × magnification). Abbreviation: sLS/BS, single-labelled surface/bone surface; dLS/BS, double-labelled surface/bone surface; MAR, mineral apposition rate; BSL, baseline; SHAM, sham-operated; OVX, ovariectomised; AT, ovariectomised + annatto tocotrienol 60 mg/kg; SEAT, ovariectomised + annatto tocotrienol 60 mg/kg formulated with self-emulsifying system; RAL, ovariectomised + raloxifene 1 mg/kg group.

Figure 2 shows representative fluorescent photomicrographs of the trabecular bone at distal femoral metaphyses. More sLS was observed in the OVX group compared to the BSL and SHAM groups. More dLS was observed in the AT, SEAT and RAL groups. Analysis of bone dynamic histomorphometric parameters (Fig. 2A-E) showed that ovariectomy increased sLS/BS (p=0.002 and p<0.001) and decreased MAR (p<0.001 and p=0.001) of the OVX group significantly compared to the BSL and SHAM group. Significant reduction in dLS/BS (p=0.001), MS/BS (p=0.006) and BFR/BS (p=0.001) were also observed in the OVX group compared to the SHAM group. Raloxifene (p<0.001), SEAT (p<0.001) and AT (p=0.005) showed lower sLS/BS value versus the OVX group. AT (p=0.018) and SEAT (p=0.036) increased MAR significantly versus the OVX group. Only SEAT showed a significant increase in dLS/BS (p=0.030) and BFR/BS (p=0.009) versus the OVX group. No significant inter-group difference was observed in MS/BS (p>0.05).

**Figure 3:**
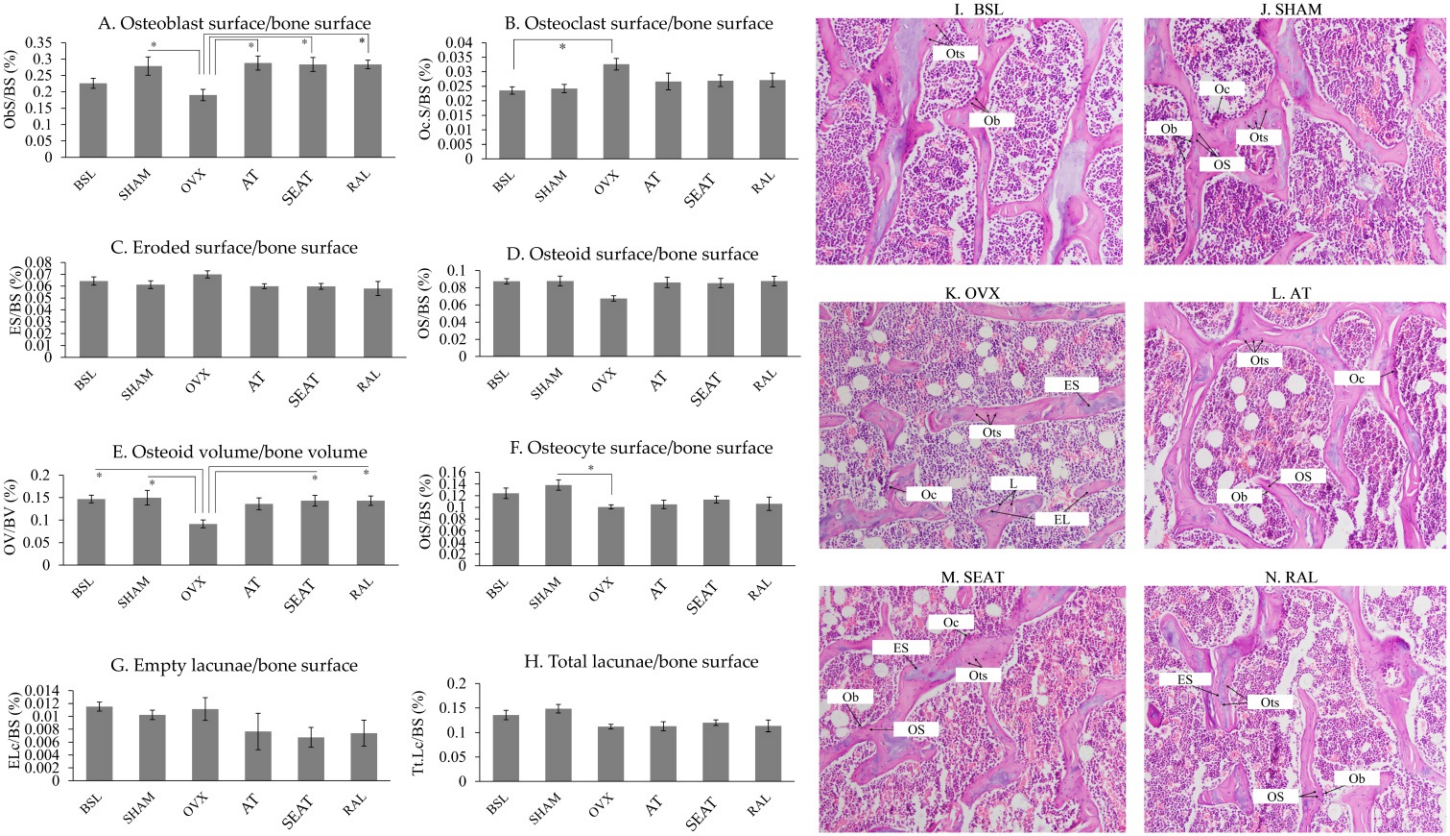
The distribution of bone cells and osteoid on the trabecular bone at the distal femur are determined via bone cellular histomorphometric indices (A-H). The data are expressed in mean ± standard error of the mean. *, ** and *** indicate statistically significant differences at p<0.05, p<0.01 and p<0.001. Photomicrograph (I-N) shows trabecular bone at the distal femur. Decalcified section (20 × magnification) shows trabecular bone (red-pink) with osteoclasts and osteoblasts surrounding the bone. Abbreviation: EL, empty lacunae; ES, eroded surface; L, lacunae; Ob, osteoblast; Oc, osteoclast; OS, osteoid surface; Ots, osteocyte; BSL, baseline; SHAM, sham-operated; OVX, ovariectomised; AT, ovariectomised + annatto tocotrienol 60 mg/kg; SEAT, ovariectomised + annatto tocotrienol 60 mg/kg formulated with self-emulsifying system; RAL, ovariectomised + raloxifene 1 mg/kg group.

Figure 3 shows H&E-stained micrographs of trabecular bone at the distal femoral metaphyses. Fewer osteoblasts were noticeable in the OVX group than the other groups. Bone cellular index analysis (Fig. 3A-H) indicated that ovariectomy increased OcS/BS (p=0.044) and decreased OV/BV (p=0.027) significantly in the OVX group compared to the BSL group. Significant reductions in ObS/BS (p=0.041), OV/BV (p=0.017) and OtS/BS (p=0.029) were observed in the OVX group versus the SHAM group. Treatment with AT (p=0.019) and SEAT (p=0.027) significantly increased ObS/BS. Only SEAT increased OV/BV significantly versus the OVX group (p=0.045). ObS/BS (p=0.027) and OV/BV (p=0.045) also increased significantly in the RAL group than the OVX group. Other cellular parameters, i.e. OcB/BS, ES/BS, OS/BS, OtS/BS, ELc/BS and TtL/BS, were similar among the study groups (p>0.05).

**Figure 4:**
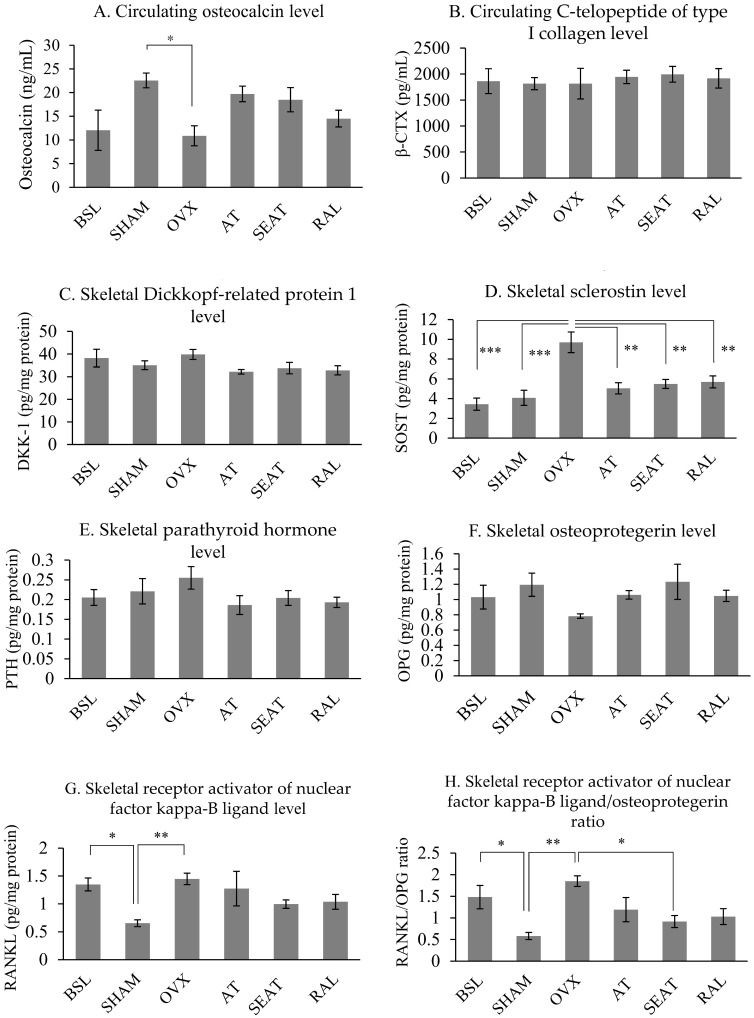
Circulating bone remodelling markers (A, B) and osteocyte-related factors influencing bone remodelling in rats (C-H). The data are expressed in mean ± standard error of the mean*, ** and *** indicate statistically significant differences at p<0.05, p<0.01 and p<0.001. Abbreviation: BSL, baseline; SHAM, sham-operated; OVX, ovariectomised; AT, ovariectomised + annatto tocotrienol 60 mg/kg; SEAT, ovariectomised + annatto tocotrienol 60 mg/kg formulated with self-emulsifying system; RAL, ovariectomised + raloxifene 1 mg/kg group.

The circulating osteocalcin level of the OVX group was significantly lower than the SHAM groups (p=0.026). However, circulating osteocalcin level was not significantly different among other study groups (p>0.05). No significant differences in circulating β-CTX level were observed in all study groups (p>0.05) (Fig. 4A&B). Ovariectomy increased skeletal protein expression of SOST (p*<*0.001), sRANKL (p=0.002) and RANKL/OPG ratio (p=0.001) significantly compared to the SHAM group. Raloxifene (p=0.004), SEAT (p=0.003) and AT (p=0.001) significantly decreased the SOST level versus the OVX group. Only SEAT decreased RANKL/OPG ratio significantly versus the OVX group (p=0.021). However, no significant differences were observed in DKK-1, PTH and OPG parameters among all the study groups (p>0.05) (Fig. 4C-H).

## Discussion

This study showed that AT and SEAT improved bone mineralisation and osteoblast number in rats with prolonged oestrogen deficiency by reducing the sclerostin level in the bone. SEAT was more effective in the skeletal anabolic effects, and it reduced RANKL/OPG ratio significantly in these rats. The current study results complement the previous findings that the self-emulsifying system improved the bioavailability of AT and its effects on bone microstructure and strength 29.

Bone histomorphometry is a two-dimensional tool to quantify bone cellular features (via static/cellular indices) and the remodelling process (via dynamic indices) 33. The current study found that ovariectomy-induced oestrogen deficiency decreased active mineral deposition, indicated by a significant increase in sLS/BS and a decrease in dLS/BS, MAR, MS/BS and BFR. This observation is in line with previous studies demonstrating the destruction of skeletal microarchitecture 34 and a lack of active mineralisation on the bone 35, 36. Ovariectomy also led to decreased osteoblast activity as indicated by a significant reduction in ObS/BS and OtS/BS and decreased matrix synthesis indicated by reduced OV/BV. These events adversely affect bone integrity and subsequently lead to osteoporosis. These findings agreed with the previous observation on cellular indices of ovariectomised rats 24. However, changes in osteoclast-related parameters were not apparent in this study. Previous studies mostly used ovariectomised growing female rats, but our studies used aged ovariectomised female rats. Age difference could contribute to the difference in bone cellular indices. Similar changes were reflected in circulating bone remodelling markers, whereby osteocalcin level was lower in the OVX group than the SHAM group. In studying ageing female rats, Netto et al. found a progressive decline in serum osteocalcin and CTX from 26 to 56 weeks of age, suggesting a slow bone remodelling 37. However, β-CTX1 (bone resorption marker) level in the current study was not significantly altered by ovariectomy.

In this study, treatment with both AT and SEAT increased sLS/BS and MAR but only SEAT increased dLS/BS and BFR/BS in ovariectomised rats. Based on the fluorescent calcein labelling, the presence of dLS reflects mineral deposition on the bone surface over seven days, while the sLS indicates a lack of mineral deposition because the two calcein labels superimpose on each other. Since data on the skeletal effects of SEAT are limited, we compared the current results with preventive models using unformulated tocotrienol. Similar findings were observed by Chin et al., whereby AT (60 mg/kg b.w.) reduced sLS/BS and increased dLS/BS, MS/BS, MAR and BFR/BS when administered concurrently with lovastatin 23. In the metabolic syndrome-induced bone loss model, AT (60 mg/kg b.w.) reduced sLS/BS and increased MAR 38. The rise in bone formation and mineralisation caused by AT suggests that it could increase osteoblastic activity. A significant increase of ObS/BS and OtS/BS in AT-treated groups showed that AT could also exert its anabolic action by increasing the number of functioning osteoblasts and osteocytes. The supplemented group also had higher OV/BV, indicating AT increased matrix synthesis by osteoblasts. These bone cellular histomorphometric changes caused by tocotrienol in ovariectomised rats have been demonstrated previously 24, 34. Therefore, the current findings suggest that AT preserves bone microarchitecture by stimulating proliferation and activity osteoblasts, so more new bone is synthesised. Despite this, no significant differences in circulating remodelling markers were observed between the treatment groups and the OVX group. Changes in bone remodelling might be transient, so they could not be captured by circulating bone remodelling markers.

The molecular mechanisms through which oestrogen deficiency stimulates bone resorption are well understood. RANKL is upregulated by oestrogen deficiency, leading to increased osteoclast recruitment and activation, as well as decreased osteoclast apoptosis 39. Oestrogen deficiency also suppresses the production of OPG by osteoblasts, skewing the RANKL/OPG ratio towards the promotion of osteoclast formation and bone resorption 40. Our current study showed that soluble RANKL protein expression in the bone tissue increased significantly in the OVX group, but the OPG level was not altered. Thus, an increase in RANKL/OPG ratio was observed in the OVX group. Oestrogen deficiency is also associated with decreased calcium absorption efficiency, which may elevate PTH in response to the drop of circulating calcium level 41. PTH also suppresses OPG expression in early osteoblasts while elevates RANKL expression in mature osteoblasts 42. However, PTH level was measured in the bone but not in the blood in this study, and its skeletal level was not significantly affected by OVX. SOST 43 and DKK-1 44 inhibit bone formation by antagonising the Wnt/β-catenin signalling pathway, which stimulates osteoblast activity and attenuates osteoclast function. Clinically, a high SOST level is a strong and independent risk factor for osteoporotic fractures among postmenopausal women 45. Increased SOST level was observed in the OVX group compared to the SHAM group in this study. Treatment with AT and SEAT suppressed SOST levels in ovariectomised rats, suggesting the involvement Wnt/β-catenin pathway in the skeletal action of AT. A previous study by Chin et al. observed that AT increased the mRNA expression of β-catenin in bone tissue from orchidectomised rats 26. The current study also showed that only SEAT decreased the RANKL/OPG ratio significantly. This observation agrees with a human clinical trial study showing a significant decrease in serum sRANKL and soluble RANKL/OPG ratio among postmenopausal women with osteopenia after twelve-week tocotrienol administration 46. Others reported that AT could increase expression of genes coding for alkaline phosphatase, collagen type I alpha 1 and osteopontin and a reduction RANKL mRNA expression in orchidectomised rats 26. In rats with metabolic syndrome, AT lowered RANKL, SOST, DKK-1 and fibroblast growth factor-23 level 47. In contrast, we did not observe significant changes in these proteins between the OVX group and the treatment groups. The distinction might be due to the models used, whereby the rats of this study developed significant bone loss before supplementation, whereas others supplemented the rats upon initiation of bone loss.

Our previous study demonstrated that improvements in bone structure, calcium content and strength were almost similar between AT and SEAT, despite a 4-fold increase in circulating delta-tocotrienol level 29. It is postulated that a therapeutic threshold for the skeletal effects of AT might exist, whereby further increasing its bioavailability or doses would not produce better skeletal effects. Since AT and SEAT could reverse bone loss in rats with oestrogen deficiency, and the difference in skeletal effects between these two formulations was marginal, we propose that a self-emulsifying system would allow lower doses of AT to be used in osteoporosis treatment and achieve the same effects of unformulated AT at higher doses. Nevertheless, the skeletal efficacy of this approach needs to be validated in future studies. Using a lower amount of AT in the formulation without compromising its effects is more economical. It will enhance the affordability of AT and allow this nutraceutical to be accessible to a broader population.

In this study, some limitations need to be addressed. Tartrate-resistance acid phosphatase staining was not performed for osteoclasts in this study. Instead, the identification of osteoclasts throughout the histology sections was dependent on cell morphology. We also did not validate the anti-inflammatory activities of AT, which could partially explain its bone protective action. Nevertheless, this study is among the few that highlight the use of a self-emulsifying system in improving the clinical application of AT, particularly as an anti-osteoporosis agent.

## Conclusion

In conclusion, AT exerts skeletal anabolic action in rats with oestrogen deficiency by improving bone formation. The skeletal effects of AT could be exerted by suppressing the skeletal SOST level. SEAT exerts slightly better anabolic actions and also reduced the skeletal RANKL/OPG ratio. Further studies are needed to illustrate the regulatory mechanism of AT on the SOST level.
